# The Novel Use of an External Oblique Nerve Catheter After Open Cholecystectomy

**DOI:** 10.7759/cureus.13580

**Published:** 2021-02-26

**Authors:** Brendan O'Donovan, Brian Martin

**Affiliations:** 1 Anesthesiology, University of Massachusetts Medical School-Baystate, Springfield, USA

**Keywords:** open cholecystectomy, external oblique nerve block, tap block, abdominal wall innervation, external oblique nerve catheter, quadratus lumborum block

## Abstract

Open cholecystectomy is a painful procedure and requires a well-rounded multimodal approach for successful postoperative analgesia. A thoracic epidural is known to provide reliable pain relief for upper abdominal surgical procedures. However, in patients for whom an epidural is contraindicated, an alternative regional technique may be sought. This case discusses the novel use of an external oblique catheter after open cholecystectomy.

## Introduction

Open cholecystectomy is a potentially painful operation. A thoracic epidural is known to provide reliable pain relief for upper abdominal surgery [1]. When an epidural placement is contraindicated, however, pain management can be difficult. The Kocher incision is a subcostal incision used for open cholecystectomy [2]. This incision encroaches on the T6-T9 dermatomes on the right subcostal abdominal region [3].

A clear understanding of the innervation of the abdominal wall is necessary to utilize regional anesthesia as part of a multimodal analgesic approach for post-surgical patients. The abdominal wall is supplied by the intercostal nerves (T6-T11), the subcostal nerve (T12), and the ilioinguinal/iliohypogastric nerves (L1) [4]. These nerves are derived from their respective anterior rami of the T6-L1 spinal nerves. Around the midaxillary line, the lateral cutaneous branch leaves its respective intercostal nerve and travels superficially, piercing the external intercostal muscle and/or external oblique muscle to supply sensation to the lateral abdominal wall. The lateral cutaneous nerve divides into anterior and posterior branches [5]. The anterior branch extends anteriorly as far as the rectus abdominis margin, and the posterior branch extends posteriorly to supply the skin overlying the latissimus dorsi. The intercostal nerve continues its course in the transversus abdominis plane (TAP) until it reaches the rectus abdominis muscle and divides into the anterior cutaneous branch, which supplies the skin of the midline abdomen.

The traditional lateral TAP block is performed at the midpoint between the iliac crest and the subcostal margin in the midaxillary line [4]. Cadaveric injection of dye using this approach showed involvement of T11 and T12 100% of the time, L1 93% of the time, and T10 50% of the time [6]. A lateral TAP block may not reach a dermatome high enough to benefit a patient after an open cholecystectomy. 

The oblique subcostal TAP block is performed on the anterior abdominal wall parallel to the subcostal margin with a needle entry point near the xiphoid process [4]. Chen et al. studied the extent of analgesia after the oblique subcostal TAP block [7]. The study showed dermatomal coverage of T7-T12 100% of the time, T6 75% of the time, and L1 50% of the time. The study also shed light on the fact that the oblique subcostal TAP block primarily anesthetizes the mid-abdomen; however, it leaves the lateral abdomen unblocked. The subcostal TAP block would be preferred over the lateral TAP block for open cholecystectomy, however, it may not completely cover the surgical site due to the lack of lateral abdominal wall coverage.

The quadratus lumborum block (QLB) is performed proximal to the branching of the lateral cutaneous nerve and near the quadratus lumborum muscle, which is closer to the anterior rami of the spinal nerves compared to the TAP block. The quadratus lumborum is encased by the thoracolumbar fascia (TLF), and local anesthetic injection into the TLF is thought to be responsible for the craniocaudal spread as well as spread to the paravertebral space [8]. The TLF contains mechanoreceptors and sympathetic fibers. Blockade of these sympathetic fibers and spread to the paravertebral space are thought to contribute to the visceral analgesia achieved with the QLB. Comparatively, the TAP block solely causes somatic analgesia and does not result in visceral analgesia [9]. The current literature describes four different variants of the QLB: QL1/lateral, QL2/posterior, QL3/anterior, and QL4/intramuscular. The QLB provides reliable dermatomal coverage of T7-L1, however, there are reports of spread as high as T4 and as low as L3 [10]. Perhaps, the QLB would be a preferred technique compared to both the lateral and subcostal TAP blocks for analgesia after an open cholecystectomy due to its extent of dermatomal coverage, the addition of visceral analgesia, and its ability to provide both anterior and lateral abdominal wall coverage.

Although there have been no studies comparing the TAP block versus the QLB for open cholecystectomy, two recent studies have compared the techniques for laparoscopic cholecystectomy. Baytar et al. compared the effectiveness of the subcostal TAP block and the posterior QLB for postoperative analgesia after laparoscopic cholecystectomy [11]. The study concluded that both techniques produced similar reductions in postoperative pain scores and opioid consumption. The study stated that the performance of the QLB took eight minutes longer than the subcostal TAP block and that they recommend the subcostal TAP block due to easier application of the block. Weheba et al. produced a similar study with different results [12]. Like the Baytar study, this trial compared the efficacy of the subcostal TAP block and the posterior QLB in providing postoperative analgesia after laparoscopic cholecystectomy. Although the Weheba study showed similar postoperative pain scores between the two groups, the QLB group had a significantly longer time to the first postoperative request for rescue analgesia and less number of patients required postoperative opioids compared to the TAP group.

## Case presentation

**Figure 1:**
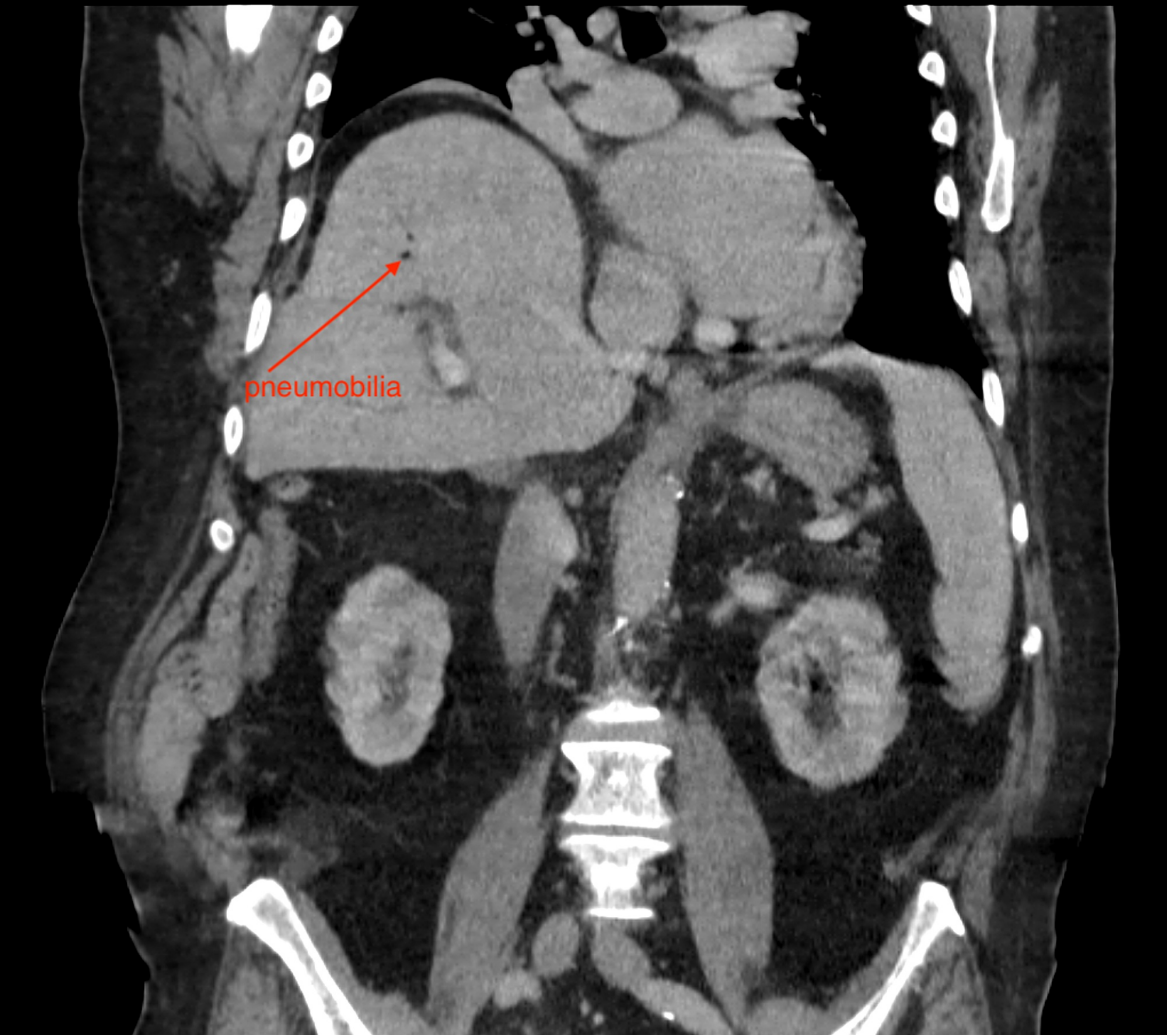
Coronal CT image showing pneumobilia

**Figure 2:**
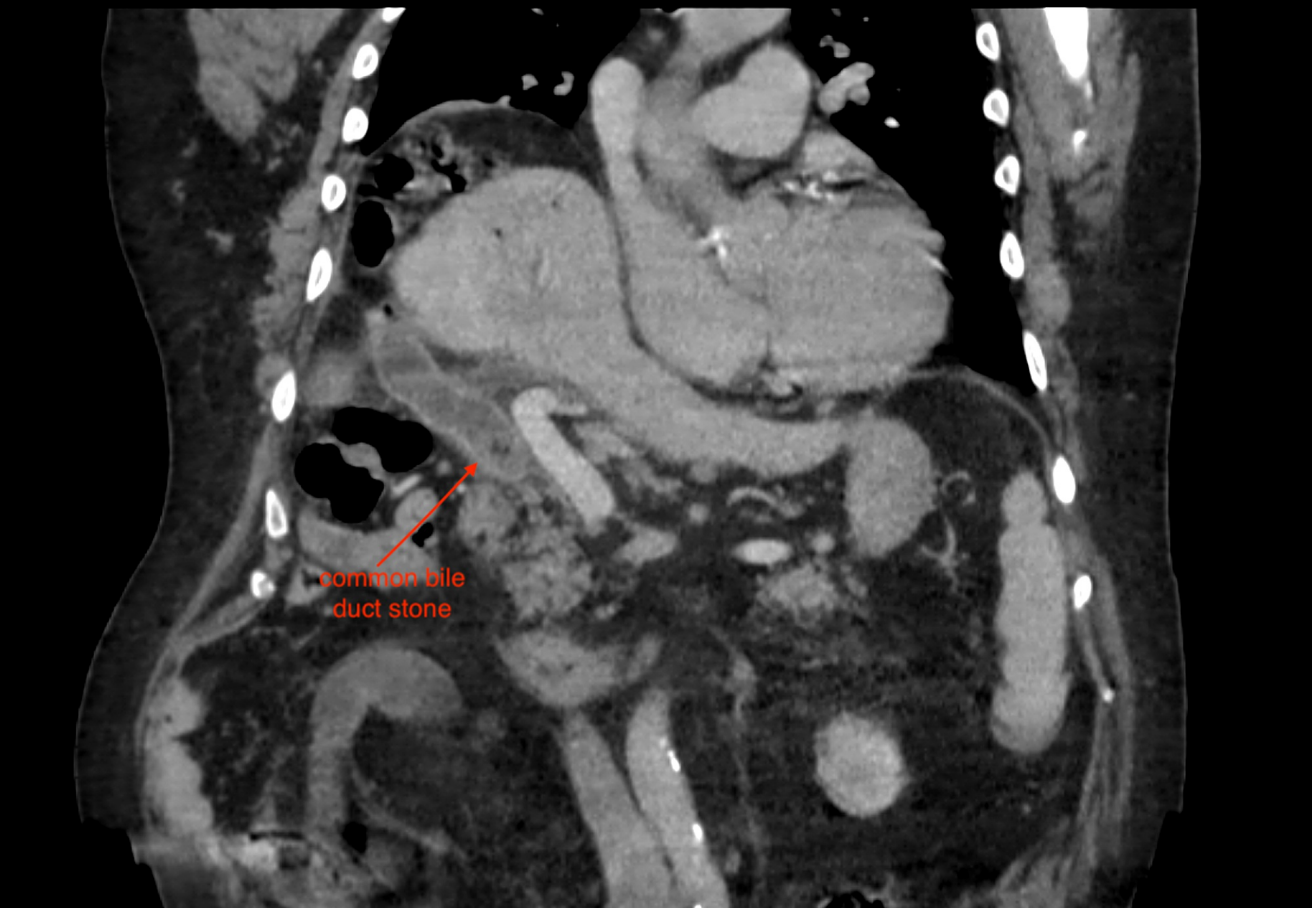
Coronal CT image showing a common bile duct stone

A 78-year-old male with a medical history of hypertension, diabetes, gout, hyperlipidemia, chronic kidney disease stage 2, and deep vein thrombosis (on Warfarin therapy) presented to an urgent care facility with signs of weakness, “dark-urine,” and jaundice. In the emergency department, he was found to be in new-onset atrial fibrillation. His initial labs were significant for leukocytosis, transaminitis, and hyperbilirubinemia. An abdominal CT showed a common bile duct stone, pneumobilia, and signs of ascending cholangitis (Figures 1, 2). He was admitted to the medicine service, started on broad-spectrum antibiotics, and gastroenterology was consulted to perform an endoscopic retrograde cholangiopancreatography (ERCP). Due to the patient’s new-onset atrial fibrillation, echocardiography was performed, showing an ejection fraction of 25-35%. He was placed on a heparin infusion due to new-onset atrial fibrillation and a history of deep venous thrombosis. An ERCP was performed on hospital day 2; however, it was unsuccessful at removing a common bile duct gallstone. The diagnosis of ascending cholangitis was made by visualization of a 2 cm common bile duct stone and pus draining from the papilla. 

The patient was taken to the operating room on hospital day 5 for a laparoscopic cholecystectomy. The surgical team converted to an open procedure by performing a right upper quadrant Kocher due to aberrant anatomy and extensive adhesions. They were unable to identify the gallbladder. The common bile duct stone was removed surgically, and a biliary T-tube was placed. As the surgical team was closing during the operation, they inquired about the placement of a nerve catheter as an epidural was contraindicated due to the patient’s need for anticoagulation for new-onset atrial fibrillation. At this point, the incision was mostly closed; however, the external oblique was still exposed. The surgical team placed a B Braun Perifix® catheter on the surface of the external oblique muscle in the right upper quadrant at the T8 dermatome. The acute pain anesthesiology service managed the nerve catheter. The patient was extubated in the operating room and was taken to the surgical intensive care unit for close monitoring. 

The acute pain service saw the patient on postoperative day 1. His pain was managed with a bupivacaine 0.1% infusion at 7cc/hr going via the nerve catheter. He was found to have 0/10 pain at rest. He attributed 3/10 pain only when his incision site was palpated and during movement. The acute pain service followed the patient until the catheter was removed on postoperative day 5. Each day the patient had virtually zero pain at rest and attributed only mild pain when his incision site was palpated or during movement. The patient’s postoperative opioid requirement was remarkably low as well. He received one dose of oxycodone 5mg on postoperative days 1 and 2, respectively. He received one dose of acetaminophen 650mg on postoperative days 1 and 4. He received no other analgesic medication in his acute postoperative course. 

## Discussion

The use of an external oblique nerve catheter for postoperative analgesia has not been described yet in the literature. Due to the low pain scores achieved and minimal narcotic use postoperatively, we hypothesize that we successfully anesthetized the lateral cutaneous branch and the anterior cutaneous branch of the thoracoabdominal nerves. Recently, there have been several publications describing external oblique nerve blocks to control post-operative pain after abdominal surgery. 

In 2018 Hamilton et al. proposed that injecting a local anesthetic into a thoracic fascial plane can block the lateral cutaneous branches from T7-T11 providing adequate analgesia for lateral abdominal surgery [13]. Hamilton et al. solidified their hypothesis by a cadaver study that looked at the spread of dye after an external oblique fascial plane block [14]. The block was performed at the sixth intercostal space in the midclavicular line, and the needle tip was either deep or superficial to the external oblique muscle. The study showed that injection of 20cc of dye effectively covered the anterior divisions of the lateral cutaneous branches of the thoracoabdominal nerves from T6-T10. 

In 2019, Tulgar et al. described a novel nerve block for upper abdominal wall analgesia, which they named a TAPA block (blockage of thoracoabdominal nerves through perichondral approach) [15]. This block is performed at the costal margin where the 9th and 10th ribs meet. A linear transducer is placed on the costochondral angle in the sagittal plane. The authors injected 20cc of injectate between the chondrium’s upper surface and the external oblique and 20cc of injectate between the chondrium’s lower surface and the transversus abdominis muscle [16]. The authors state that the TAPA block provides analgesia from T5-T12. They hypothesize that the TAPA block anesthetizes the lateral cutaneous branch as well as the anterior cutaneous branch providing analgesia from the anterior axillary line to mid-abdomen from T7-T12. They believe the block only reaches the lateral cutaneous branch of T5-T6 as these patients had pinprick sensation 4-5cm lateral to the sternum.

We believe our nerve catheter provided excellent postoperative analgesia based on a similar principle that Hamilton and Tulgar have recently discovered. As our nerve catheter was placed superficial to the external oblique muscle, we believe we anesthetized the lateral cutaneous branch as well as the anterior cutaneous branch of the thoracoabdominal nerves from T6-T9. We hypothesized that as the lateral cutaneous branch lies above the external oblique muscle, it could be effectively blocked by a nerve catheter. Furthermore, as the patient had no midline pain postoperatively, we believe we anesthetized the anterior cutaneous branch as well. We presume the local anesthetic traveled in the subcutaneous tissue toward the midline blocking the anterior cutaneous branch overlying the rectus abdominis muscle.

Although, in our case, the nerve catheter was placed under direct surgical visualization, we believe it can be successfully placed under ultrasound guidance. Perhaps this technique would be preferred in the morbidly obese population as the ultrasound target is more superficial and accessible compared to a TAP block or a QLB. The QLB would be an effective technique for postoperative analgesia following an open cholecystectomy in the majority of patients; however, it is considered a deep regional block, and its utilization is controversial in patients on anticoagulation [8]. There is a case report describing two incidences of hematoma formation following QLB, likely due to inadvertent lumbar artery puncture [17]. In addition, the QLB is considered an advanced regional technique that may be difficult to perform in inexperienced hands, whereas the superficial nature of the external oblique nerve catheter makes it easier to perform.

## Conclusions

Pain control following an open cholecystectomy can be difficult to manage, especially when an epidural is contraindicated. A feasible option for pain control may be a subcostal external oblique catheter. We hypothesize we were able to anesthetize the lateral cutaneous branch as well as the anterior cutaneous branch of the thoracoabdominal nerves from T6-T9. Future studies are required to determine the effectiveness of external oblique nerve catheters.

## References

[1] Manion SC, Brennan TJ, Riou B (2011). Thoracic epidural analgesia and acute pain management. Anesthesiology.

[2] Jones MW, Deppen JG (2021). Open cholecystectomy. StatPearls [Internet].

[3] Fischer HB, Pinnock CA (2004). Peripheral nerve blocks. Fundamentals of Regional Anaesthesia.

[4] Statzer N, Cummings KC 3rd (2018). Transversus abdominis plane blocks. Adv Anesth.

[5] Court C, Vialle R, Lepeintre JF, Tadié M (2005). The thoracoabdominal intercostal nerves: an anatomical study for their use in neurotization. Surg Radiol Anat.

[6] Tran TM, Ivanusic JJ, Hebbard P, Barrington MJ (2009). Determination of spread of injectate after ultrasound-guided transversus abdominis plane block: a cadaveric study. Br J Anaesth.

[7] Chen Y, Shi K, Xia Y, Zhang X, Papadimos TJ, Xu X, Wang Q (2018). Sensory assessment and regression rate of bilateral oblique subcostal transversus abdominis plane block in volunteers. Reg Anesth Pain Med.

[8] Dhanjal S, Tonder S (2020). Quadratus lumborum block. StatPearls [Internet].

[9] Elsharkawy Elsharkawy, Hesham Hesham, and Thomas F. Bendtsen (2021). Ultrasound-Guided Transversus Abdominis Plane and Quadratus Lumborum Nerve Blocks. NYSORA, 11 Feb.

[10] Akerman M, Pejcic N, Velickovic I (2018). A review of the quadratus lumborum block and ERAS. Front Med.

[11] Baytar C, Yilmaz C, Karasu D, Topal S (2019). Comparison of ultrasound-guided subcostal transversus abdominis plane block and quadratus lumborum block in laparoscopic cholecystectomy: a prospective, randomized, controlled clinical study. Pain Res Manag.

[12] Weheba H, Abdelsalam T, Ghareeb S, Makharita MY (2019). Posterior quadratus lumborum block versus subcostal transversus abdominis plane block in laparoscopic cholecystectomy. Int J Anesth Anesth.

[13] Hamilton DL, Manickam BP (2018). Is a thoracic fascial plane block the answer to upper abdominal wall analgesia?. Reg Anesth Pain Med.

[14] Hamilton DL, Manickam BP, Wilson MA, Abdel Meguid E (2019). External oblique fascial plane block. Reg Anesth Pain Med.

[15] Tulgar S, Senturk O, Selvi O, Balaban O, Ahiskalioğlu A, Thomas DT, Ozer Z (2019). Perichondral approach for blockage of thoracoabdominal nerves: anatomical basis and clinical experience in three cases. J Clin Anesth.

[16] Tulgar S, Ahiskalioglu A, Selvi O, Thomas DT, Ozer Z (2019). Similarities between external oblique fascial plane block and blockage of thoracoabdominal nerves through perichondral approach (TAPA). J Clin Anesth.

[17] Visoiu M, Pan S (2019). Quadratus lumborum blocks: two cases of associated hematoma. Pediatr Anesth.

